# Treatment of Idiots

**Published:** 1848-01

**Authors:** 


### TREATMENT OF IDIOTS.

We have received an exceedingly interesting pamphlet on the treatment of Idiots, the production of Mr. George Sumner, of Boston, whose letter to the Mayor of that city on Prison Discipline we had occasion to notice some months since. The remarkable fact has been established, as appears from Mr. Sumner's narrative, that idiots are susceptible of education. We are compelled by want of room to postpone a full notice of this valuable document, but shall endeavor to give the substance of it in our next number.

